# A rare case of Ralstonia mannitolilytica infection in an end stage renal patient on maintenance dialysis during municipal water contamination

**DOI:** 10.12669/pjms.334.13112

**Published:** 2017

**Authors:** Christopher Thiam Seong Lim, Sut Enn Lee

**Affiliations:** 1 Christopher Thiam Seong Lim, Unit of Nephrology, Department of Medicine, Faculty of Medicine and Health Sciences, Universiti Putra, Malaysia; 2 Sut Enn Lee, Department of Medicine, Kajang Hospital, Selangor, Malaysia

**Keywords:** Dialysis, End stage renal failure, Ralstonia mannitolilytica

## Abstract

Ralstonia mannitolilytica is a gram negative soil bacterium. Ralstonia infection though rare, has become the emerging nosocomial pathogens in hospital settings. Various clinical manifestations had been described as well as the mode of transmission. Despite its low virulence factor, it is able to survive under harsh condition and this may potentially cause significant morbidity and mortality especially in immunocompromised patients. Outbreak of Ralstonia mannitolilytica infections in the hospital are typically associated with contaminated medical supplies or instruments. We described here a case of Ralstonia mannitolilytica infection in a dialysis patient that occurred during the municipal reservoir water contamination crisis. In this report, we will also describe the behaviour of Ralstonia genus and its 4 main species, namely R. pickettii, R. solanacearum, R. insidiosa, and R. mannitolilytica and the choices of antibiotic therapy based on literature review.

## INTRODUCTION

Proteobacteria is the major taxonomic category of the gram negative bacteria.^1^ *Ralstonia* is a lesser known member of the group. Nevertheless, they are the emerging nosocomial pathogens in hospital settings, particularly associated with immunocompromised patients. Clinical conditions associated with *Ralstonia* spp. range from minor respiratory infections to severe invasive infections such as sepsis or meningitis.^2^ This genus can exist under harsh condition. It is able to survive disinfectants and thrives best in moist environment, growing in various water sources.^3^ There is limited literature on the surveillance and monitoring of the antibiotic resistance of these bacteria. They are largely limited to a few clinical strains without accounting for genotypic or phenotypic diversity.^2^ We illustrate here a case of a successfully treated *Ralstonia* infection in an end stage renal failure (ESRF) patient on regular dialysis via a permanent catheter.

## CASE HISTORY

Our case involved a 65 year old ESRF lady with underlying hypertension, diabetes mellitus and ischaemic heart disease who received regular dialysis thrice a week in her usual satellite renal dialysis centre. She has been receiving dialysis via a left sided internal jugular cuffed permanent catheter, which was inserted in 2015 due to difficult vascular access in her arms. Proper catheter dressing with mupirocin ointment application at exit site was carried out after each of her dialysis sessions. She has never experienced any dialysis access related infection before.

On this occasion, she encountered an acute onset of high grade febrile illness during one of her regular dialysis sessions, which developed approximately 90 minutes upon initiation of dialysis. It was associated with chills, rigors and malaise. The timing of her infection coincided with the contamination of the river feeding the reservoir plant which supplied the dialysis water to both her dialysis center and her house. In addition there are no other patients who experience any similar symptoms as she did. The dialyzer was a reused dialyzer and it had been checked for bundle volume integrity and undergone adequate chemical disinfection. The total viable microbial counts and endotoxin level taken from various points of the reverse osmosis system were all well below the maximum allowed limit.

On examination, her blood pressure was 154/74mmHg, heart rate was 78 beats per minute and she was febrile with temperature reading of 39.5 degree Celsius. The right cuffed catheter exit site was noted to be clean and not erythematous. There was no track tenderness to suggest catheter cuff infection. She was promptly referred and admitted to the hospital with the diagnosis of cuffed catheter sepsis. Intravenous (IV) ceftazidime dose 1g OD and IV cloxacillin 500mg QID were commenced after blood cultures were taken from the catheter and peripheral site. Her total white cell count was 8.1x10^9^/L, predominantly 93%; neutrophils and C-reactive protein was 31 mg/L. Blood cultures and sensitivity from peripheral blood and lumen revealed *Ralstonia mannitolilytica* on day three, sensitive to ceftazidime and piperacillin/tazobactam; resistant against amikacin, gentamicin, meropenem and polymycin. IV ceftazidime was continued and cloxacillin was ceased. Recovery was uneventful and she was discharged well after a week of admission. She received IV ceftazidime 2g every other day at her dialysis center for a further of two weeks outpatient antibiotic treatment.

## DISCUSSION

*Ralstonia* genus is an aerobic, non fermentative, oxidase positive, gram negative bacillus.^4^ The *Ralstonia* genus consists of four species, namely *R. pickettii, R. solanacearum* (Previously *Burkholderia pickettii* and *B. solanacearum*), *R. insidiosa*, and *R. mannitolilytica. Ralstonia* is classified under subphylum of betaproteobacteria. It has unique and enormous range of metabolic diversity. It is a unique organism which can switch between different metabolic pathways. They possess both chemoautotrophs and photoautotrophs properties, allowing themselves to either utilizing light source or through oxidizing nutrient rich compound from their environment for their energy needs. This unique property enables *Ralstonia* to become durable opportunistic gram-negative bacilli.

*Ralstonia* is versatile in its biodegradable capacities and plays a role in nitrogen fixation in various types of plants, oxidizing ammonium to produce nitrite. Its unique ability makes this group commonly found in water and soil. The bacterium is abundant in the environment having been isolated from a wide array of environmental sources, including municipal drinking water supplies, bottled water, dental water supplies, hospital water supplies, space shuttle water systems, standard purified water, laboratory based high purity water systems and industrial ultra- pure or high purity water.^4^ Specifically, in hospital setting, most human *Ralstonia* cases resulted from use of contaminated solutions such as distilled water, injectable water, injectable saline or purified respiratory ampoules.^5^ It is difficult to differentiate the four *Ralstonia* species using routing hospital analysis as they have similar biochemical patterns to each other. Polymerase chain reaction (PCR) remained the best technique to identify the species specific primers.^6^ Limited literature reviews have led to dilemma as to administering full antibiotic treatment to the bacteria or to just regard it as a colonizer when there is no overt clinical infection.

*Ralstonia mannitolilytica*, a gram negative soil bacterium, is closely related to *Ralstonia pickettii*. It was previously named *Pseudomonas thomasii*.^7^ A number of *Ralstonia mannitolilytica* hospital outbreaks have been reported, this includes contaminated parenteral fluids with deionised water, contaminated saline solution prepared by hospital pharmacy. The contaminations had led to severe bacteraemia, meningitis, catheter related infection, respiratory infection, haemoperitoneum and renal transplant infection.^8^ This particular strain is also the most prevalent species to be found in cystic fibrosis patients. *Ralstonia mannitolilytica* showed resistance mostly developed against ampicillin, penem and aminoglycosides group of antibiotic. Most of the clinicians used piperacillin/tazobactam and third generation cephalosporins and complete recovery was achieved in these cases.^8^ Stelzmueller et al. had reported the lack of virulence factors in most *Ralstonia* species, therefore aggressive treatment with broad spectrum antibiotics is likely to contribute to the increasingly resistant strains.^9^

On the other hand, many factors are responsible for *Ralstonia* infection. *Ralstonia* spp. has the ability to pass through 0.2 micrometer filters that are used for the sterilisation of many medical products, such as saline solution.^8^ Such permeability will means that it can potential pass through dialysis reverse osmosis membrane or even dialyzer. It has been shown to survive in chlorhexidine with 0.05% aqueous solution. This disinfectant is commonly used as topical antiseptic in venous catheter related procedures. Besides, recent polluted water in municipal treatment plant may have contributed to the emergence of this uncommon opportunistic infection.

## CONCLUSION

Despite *Ralstonia* low virulence factor, it may still potentially inflict harmful damage in the immunocompromised patients. *Ralstonia* contamination should always be kept in mind and considered, especially in ESRF patient when no other source of infection is identified as early targeted antibiotic is essential in treating this opportunistic infection. High clinical suspicion with close collaboration of laboratory system and the public health system are important to assist in prompt source identification, administering proper treatment and preventative measures.

### Author’s contribution

**LSE:** Initial draft and editing of manuscript.

**CLTS:** Manuscript writing, review and final approval of manuscript. Takes the responsibility and is accountable for all aspects of the work in ensuring that questions related to the accuracy or integrity of any part of the work.

## References

[1] Coenye T, Goris J, De Vos P, Vandamme P, LiPuma JJ (2003). Classification of Ralstonia pickettii-like isolates from the environment and clinical samples as Ralstonia insidiosa sp. nov. Int J Syst Evol Microbiol.

[2] Ryan MP, Adley CC (2013). The antibiotic susceptibility of water-based bacteria Ralstonia pickettii and Rasltonia insidiosa. J Med Microbiol.

[3] Verschraegen G, Claeys G, Meeus G, Delanghe M (1985). Pseudomonas pickettii as a cause of pseudobacteremia. J Clin Microbiol.

[4] Gilligan PH, Lum G, Vandamme PAR, Whittier S (2003). Manual of clinical microbiology. In Burkholderia Stenotrophomonas Ralstonia Brevundimonas Comamonas Delftia Pandoraea and Acidovorax. Manual Clin Microbiol.

[5] Roberts LA, Collignon PJ, Cramp VB, Alexander S, McFarlane AE, Graham E (1990). An Australian-wide epidemic of Pseudomonas pickettii bacteraemia due to contaminated “sterile” water for injection. Med J Aust.

[6] Ryan MP, Pembroke JT, Adley CC (2011). Differentiating the growing nosocomial infectious threats Ralstonia pickettii and Ralstonia insidiosa. Eur J Clin Microbiol Infect.

[7] De Baere T, Steyaert S, Wauters G, Des Vos P, Goris J, Coenye T (2001). Classification of Ralstonia pickettii biovar 3/'thomasii'strains (Pickett 1994) and of new isolates related to nosocomial recurrent meningitis as Ralstonia mannitolytica sp. Int J Syst Evol Microbiol.

[8] Ryan MP, Adley CC (2014). Ralstonia spp emerging global opportunistic pathogens. Eur J Clin Microbiol Infect.

[9] Stelzmueller I, Biebl M, Wiesmayr S, Eller M, Hoeller E, Fille M (2006). Ralstonia pickettii-innocent bystander or a potential threat?. Clin Microbiol Infect.

